# Brightness and Darkness as Perceptual Dimensions

**DOI:** 10.1371/journal.pcbi.0030179

**Published:** 2007-10-19

**Authors:** Tony Vladusich, Marcel P Lucassen, Frans W Cornelissen

**Affiliations:** 1 Laboratory of Experimental Ophthalmology & BCN NeuroImaging Centre, School of Behavioural and Cognitive Neurosciences, University Medical Centre Groningen, University of Groningen, Groningen, The Netherlands; 2 Department of Human Interfaces, TNO Human Factors, Soesterberg, The Netherlands; University College London, United Kingdom

## Abstract

A common-sense assumption concerning visual perception states that brightness and darkness cannot coexist at a given spatial location. One corollary of this assumption is that achromatic colors, or perceived grey shades, are contained in a one-dimensional (1-D) space varying from bright to dark. The results of many previous psychophysical studies suggest, by contrast, that achromatic colors are represented as points in a color space composed of two or more perceptual dimensions. The nature of these perceptual dimensions, however, presently remains unclear. Here we provide direct evidence that brightness and darkness form the dimensions of a two-dimensional (2-D) achromatic color space. This color space may play a role in the representation of object surfaces viewed against natural backgrounds, which simultaneously induce both brightness and darkness signals. Our 2-D model generalizes to the chromatic dimensions of color perception, indicating that redness and greenness (blueness and yellowness) also form perceptual dimensions. Collectively, these findings suggest that human color space is composed of six dimensions, rather than the conventional three.

## Author Summary

Vision scientists have long adhered to the classic opponent-coding theory of vision, which states that bright–dark, red–green, and blue–yellow form mutually exclusive color pairs. According to this theory, it is not possible to see both brightness and darkness at a single spatial location, or an extended set of locations, such as a uniform surface. One corollary of this statement is that all perceivable grey shades vary along a continuum from bright to dark. At first glance, the notion that brightness and darkness cannot coexist on a single surface accords with our common-sense notion that a given grey shade cannot be simultaneously both brighter and darker than any other grey shade. The results presented here suggest that this common-sense notion is not supported by experimental data. Our results imply that a given grey shade can indeed be simultaneously brighter and darker than another grey shade. This seemingly paradoxical conclusion arises naturally if one assumes that brightness and darkness constitute the dimensions of a two-dimensional perceptual space in which points represent grey shades. Our results may encourage scientists working in related fields to question the assumption that perceptual variables, rather than sensory variables, are encoded in opponent pairs.

## Introduction

**Figure 1:**
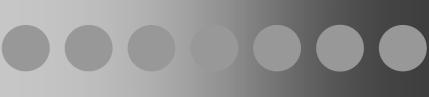
Brightness and Darkness Induction Viewed from left to right, the grey disks appear to vary from dark to bright, even though they share the same luminance value. This *induction effect* arises because the background luminance varies along a gradient, leading to a change in the polarity and magnitude of contrasts formed against the disks. Brightness refers to the perceived luminance, or grey shade, of the disk when the luminance of the disk is greater than that of the background. Conversely, darkness is defined as the perceived luminance of the disk when the luminance of the disk is less than that of the background. The conventional way of thinking about this perceptual effect is that darkness is simply the negative of brightness, meaning that all the grey shades above are contained in a 1-D continuum, like real numbers along a number line.

It is well-known that our perception of achromatic colors, or grey shades, depends on the contrast between adjacent surfaces [1]. A nearby surface induces either brightness or darkness into the target depending on the polarity of the inducing contrast (Figure 1). Brightness is perceived if the target region has higher luminance than the background (increment), whereas darkness is perceived if the target has lower luminance (decrement). It is also known that brightness or darkness is proportional to the contrast magnitude at the inducing luminance edge [1]. In more complex or naturalistic displays, both brightness and darkness may be simultaneously induced on a single surface. Some computational models of achromatic color perception [2–5] posit that brightness and darkness subtract to determine the perceived grey shade. This is equivalent to stating that bright and dark constitute the endpoints of a one-dimensional (1-D) achromatic color space containing all possible grey shades.

The 1-D computational model is contradicted by the findings of several psychophysical studies in which subjects attempt to match achromatic colors associated with different image regions (reviewed in [6]). In a typical achromatic color-matching experiment, subjects adjust the luminance of a matching surface such that the appearance of the surface matches the appearance of a reference surface. Many researchers have observed that, when such matches are completed, residual differences in the color appearance of reference and matching surfaces often persist. Such residual unmatched differences are consistent with a computational model in which achromatic color space is composed of two or more dimensions [6–8]. According to one such model [6,7], the achromatic color space is composed of one dimension corresponding to surface reflectance and the other dimension corresponding to illumination intensity. One key problem with this model is that it assumes that subjects can independently estimate reflectance and illumination, even though the eye receives only a mixture of these two information sources—a difficult, though not insurmountable, problem known as color constancy [9,10]. More immediately, it remains unclear how such a model can explain any difficulty subjects may have in matching achromatic colors in flat, computer-generated displays. As computer screens only emit light, it becomes difficult or impossible to meaningfully parse the visual display into reflectance and illumination components (just as, for example, the impression of depth is critically impoverished in the absence of binocular disparity).

The present work represents an alternative approach to identifying the dimensions of achromatic color space. Our approach is motivated by the possibility, foreshadowed above, that brightness and darkness may themselves constitute perceptual dimensions [8]. As detailed in the Discussion section, this approach offers certain advantages over the reflectance–illumination theory of achromatic color representation [6,7], including neural plausibility. Indeed, attempts have previously been made [8,11,12] to link perceived brightness and darkness to the respective properties of the parallel bright (ON) and dark (OFF) visual pathways. Although the ON and OFF pathways run in parallel from the primate retina to the second processing area (V2) of visual cortex [13], direct evidence linking them to brightness and darkness perception is lacking [14].

**Figure 2:**
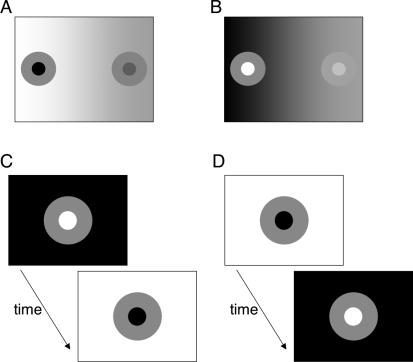
Stimuli Used in the Experiments We are interested in the question of how to describe, for example, the achromatic color percept of a ring surrounded by a dark disk inducing brightness and a white background inducing darkness. Previous work, summarized in the main text, suggests that achromatic color space is composed of two or more dimensions. Here we argue that the achromatic colors of the above rings are described by separate brightness and darkness dimensions. (A) Experiment One. Stimuli composed of a background brighter than both the reference (left side) and matching (right side) rings. A horizontal luminance gradient was rendered along the midline of the background such that the contrast formed by disk and background was different on the two sides (the gradient did not extend to the rings). Note that the contrast formed by the ring and disk on the reference side was of the opposite polarity to the contrast formed by the ring and background. (B) Similar to (A) except that the background was darker than the rings, whereas the disks were brighter than the ring. In each stimulus, the background and central disk induced brightness or darkness into the rings by means of simultaneous contrast. (C,D) Experiment Two. The polarity relationships reversed between successive presentations of reference and matching displays. See text for details of the experiments.

Here we endeavor to directly test the hypothesis that brightness and darkness form the perceptual dimensions of achromatic color space. We devise some simple visual displays in order to demonstrate that brightness and darkness signals remain separated at the highest levels of processing, rather than interacting by subtraction. In one such display, for example, a grey reference ring is bordered by a black disk and a white background (Figure 2A). The inner edge between ring and disk, and the outer edge between ring and background, have equal contrast ratios but opposite polarities. The black disk thereby induces brightness into the ring, whereas the white background induces darkness. In a second display, the grey reference ring is bordered by a white disk and a black background (Figure 2B), with the complementary consequences in terms of induction.

We model the grey shade associated with the reference ring as a point in a 2-D “grey space” formed by the induced brightness and darkness signals. Likewise, we model the grey shade associated with a matching ring—which differs from the reference ring in terms of either luminance, contrast, or both—as a point in grey space. In two psychophysical experiments, we quantify the perceptual differences between many pairs of reference and matching rings. We find that grey shades are identical only if the ring, disk, and background all possess the same luminance values in both reference and test displays (the displays are identical). The residual perceptual differences between non-identical display pairs are well-predicted by our 2-D model, even when the details of the experimental design differ markedly.

## Results

### Experiment One: Simultaneous Presentation, Variable Edge Contrast

The rationale of Experiment One is perhaps best illustrated through an example: consider the above-mentioned stimulus in which a black disk induces brightness and a white background induces darkness into a reference ring (Figure 2A). The subject attempts to adjust the luminance of the matching ring to make it appear the same grey shade as the reference ring. Now suppose that the magnitude of brightness and darkness induced into the matching ring is much less than that induced into the reference ring (Figure 2A). In other words, the matching background is light grey (rather than white) and the matching disk is dark grey (rather than black). Assuming that brightness and darkness constitute perceptual dimensions, the subject can never adjust the luminance of the matching ring to make the reference and matching rings appear exactly the same grey shade. To match the brightness dimension, for example, the subject must increase the contrast of the brightness-inducing edge in the matching display to equal the high contrast of the brightness-inducing edge in the reference display. Conversely, the subject must increase the contrast of the darkness-inducing edge in the matching display to equal the high contrast of the darkness-inducing edge in the reference display. These two options are mutually incompatible, as *increasing* the contrast of the brightness-inducing edge must necessarily *decrease* the contrast of the darkness-inducing edge, and vice versa. Thus, in attempting to decrease the mismatch in the brightness dimension, the subject inadvertently increases the mismatch in the darkness dimension, and vice versa.

The above reasoning implies that only partial color matches are possible in such a display. It further implies that the ease with which subjects can make matches will increase as the contrast difference between reference and matching displays decreases. To estimate the quality of achromatic color matches in experiment one, we had subjects rate the “possibility of making a perfect match” on a 1–10 scale, after they had completed each setting (10 = perfect match; 1 = no possible match; intermediate values = partial matches of variable quality).

**Figure 3:**
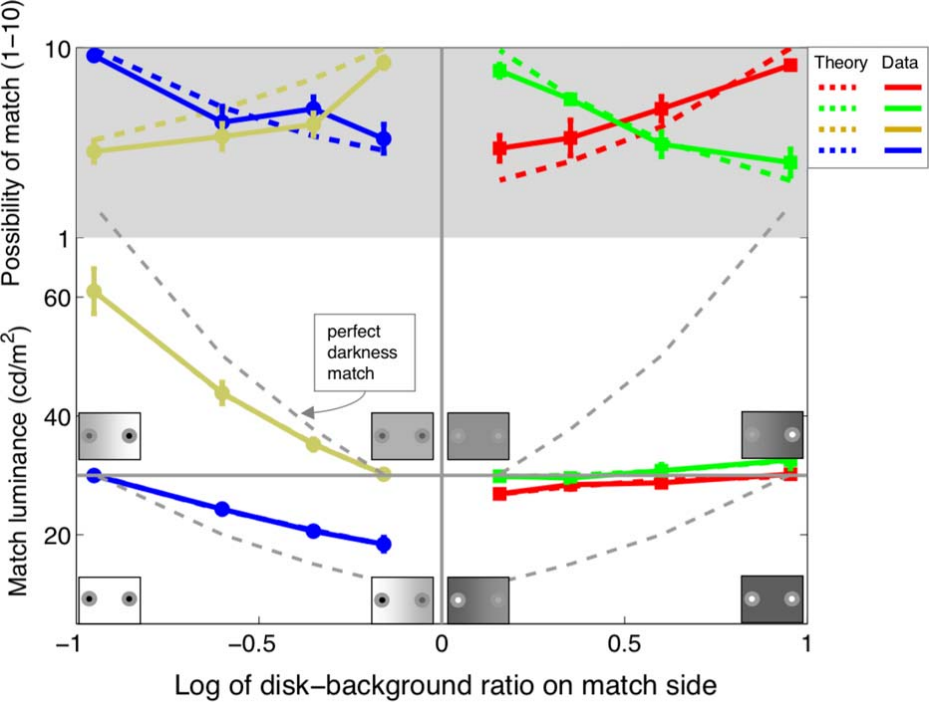
Possibility of Match Plotted against the Contrast Difference between Reference and Matching Displays Average data from six subjects (error bars indicate standard errors of the mean). With bright backgrounds (left side), subjects adjusted the luminance of the matching ring to be either much higher (yellow data points in white region) or much lower (blue data points in white region) than the luminance of the reference ring (always at 30 cd/m^2^). The dotted grey lines denote perfect darkness matches, indicating that subjects weighted darkness more heavily than brightness in this situation. With dark backgrounds (right side), subjects set the matching luminance close to the reference luminance (red and green data points in white region). In both cases, however, subjects rated matches as progressively less possible as the contrast difference between reference and matching sides of the displays increased (data points joined by continuous lines in grey region). This implies that brightness and darkness constitute dimensions of achromatic color space. We modeled this 2-D space by estimating brightness and darkness weighting factors from the luminance data (model fits are the continuous lines in the white region) and then predicting the possibility ratings from the fitted weights (dotted lines in the grey region). The model predictions agree reasonably well with the data. Symbols representing the stimuli are included to assist understanding of the data and should not be considered as realistic representations.

The results of Experiment One indicate that the possibility of making achromatic color matches declines with the contrast difference between the reference and matching rings (Figure 3). When the reference ring was bordered by a dark disk and bright background, subjects set the luminance of the matching ring closer to the luminance of the bright background. This implies that subjects placed more weight on matching darkness (induced by the brighter background) rather than brightness (induced by the darker disk). When the contrast of the darkness-inducing edge was low on the reference side of the display, for example, subjects set the luminance of the matching ring close to the luminance of the matching background in order to match this low contrast. More generally, we can say that the luminance settings fell close to theoretical predictions based on an assumption of perfect darkness matching: subjects *attempted to match the contrast of the darkness-inducing edge* across reference and matching rings, largely ignoring the contrast of the brightness-inducing edge. The results were rather different when the reference ring was bordered by a bright disk and dark background. In this case, subjects always set the luminance of the matching ring close to the luminance of the reference ring. This implies that subjects placed roughly equal weight on matching brightness and darkness in these conditions. Nonetheless, subjects rated the task of setting perfect color matches as progressively less possible as the contrast difference between match and reference displays increased, independently of the type of background.

To better understand the nature of achromatic colors, we modeled grey shades as points in a 2-D achromatic color space consisting of brightness and darkness dimensions. According to this model, subjects can perfectly match either brightness or darkness in our displays, but generally cannot match both simultaneously. We estimated the weights associated with brightness and darkness for each stimulus display by fitting the model to the luminance settings made by subjects (Materials and Methods). These fits were extremely accurate, explaining more than 96% of the variance in subjects' settings. The weights were allowed to vary with background and disk luminance to simulate gain control [4,5]: the influence of gain control was, however, quite small (∼5% of the mean weight values) and did not affect our results greatly. We then attempted to predict the possibility ratings made by subjects based on these fitted weights. Within the 2-D perceptual space, we constructed a simple dissimilarity metric based on a modified version of the city-block method (Materials and Methods). These predictions agree remarkably well with subjects' possibility ratings (Figure 3).

**Figure 4:**
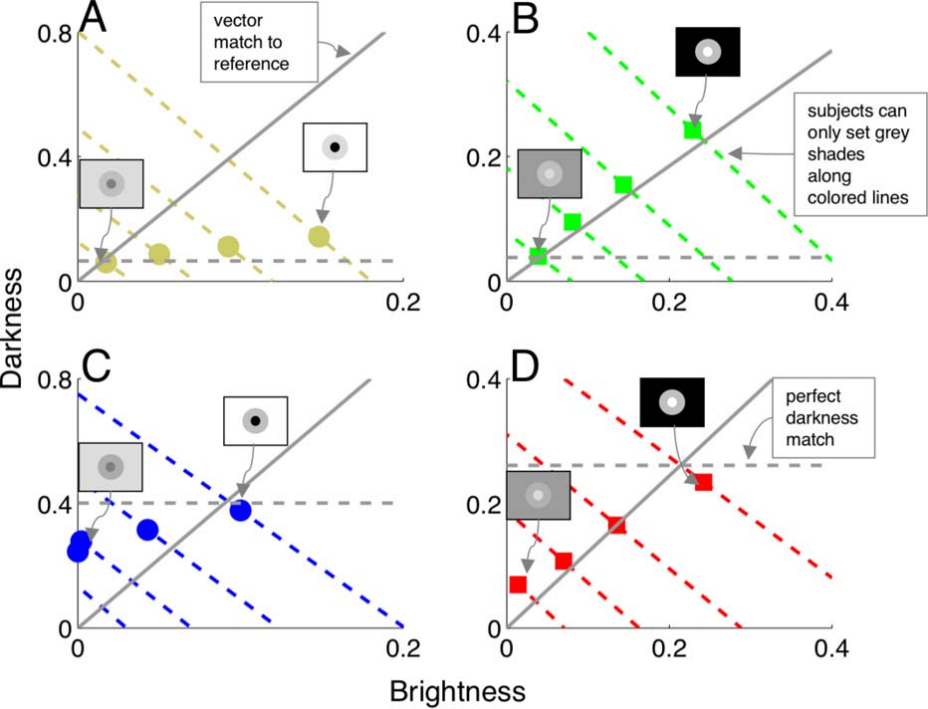
Brightness and Darkness as Perceptual Dimensions (A–D) Achromatic color space consisting of brightness and darkness dimensions. For each luminance value of the matching ring, we plot the corresponding grey shade as a point in the 2-D grey space. Horizontal dotted lines denote perfect darkness matches. Dotted colored lines represent the gamut of grey shades available in the single dimension of luminance space along which subjects can physically adjust the matching ring. Solid grey lines are the approximate vector projections of the grey shade associated with the reference ring onto all matching displays. The intersections of these projection lines with the lines-of-adjustment roughly indicate the grey shades that subjects would set if they were minimizing the vector between reference and matching rings. Subjects did not appear, however, to minimize this vector. This is particularly evident with brighter backgrounds (A,C), where subjects placed far more weight on matching the darkness component than the brightness component. The different scales for brightness and darkness in (A) and (C) provide further evidence that subjects weighted darkness more heavily than brightness.

We next plotted grey shades associated with matching rings as points in a 2-D grey space (Figure 4). We explain the impossibility of setting perfect matches by noting that subjects could only set grey values in the reference ring along the dotted colored lines (or directly along the horizontal and vertical axes, assuming that both edges share the same polarity) indicated in Figure 4. This is because adjusting the luminance of the matching ring to increase brightness (darkness) involves a simultaneous decrease in the amount of darkness (brightness). Subjects could therefore only adjust achromatic color of the reference ring along a single dimension—corresponding to luminance—within the 2-D grey space. Subjects were simply unable to tap into the rich gamut of achromatic colors available in the entire 2-D space.

The values of fitted brightness and darkness weights in our model reveal that darkness is about four times stronger than brightness in displays containing bright backgrounds (Figure 4). Why did subjects place more weight on matching the darkness dimension with bright backgrounds? We explain this behavior in terms of the well-known observation that darkness is inherently stronger than brightness [11,15,16], combined with the unequal circumferences of inner and outer ring edges. With bright backgrounds, the circumference of the outer darkness-inducing edge was three times the circumference of the inner brightness-inducing edge. Darkness was therefore weighted more heavily than its inherent value. With dark backgrounds, however, the circumference of the inner darkness-inducing edge was one-third of the circumference of the brightness-inducing edge. In this case, brightness and darkness weights were roughly equal. From these considerations, we calculate that edge weights are related to circumference by a power function with exponent ∼0.63 and that darkness is inherently about twice as strong as brightness (see Materials and Methods). This estimate is consistent with available data [11,15].

The dominance of darkness induction with bright backgrounds also indicates that possibility ratings were not based on the computation of Euclidian vectors in the 2-D space. Unlike the case with dark backgrounds (Figure 4)—where brightness and darkness weights were equally balanced—subjects did not minimize the vector between reference and matching rings. We instead found that the city-block metric [6]—in which residual color mismatches in the brightness and darkness dimensions are summed—is far more suitable for modeling the rating data (Materials and Methods). This result further implies that brightness and darkness pathways remain physically separated even at the highest processing levels (see Discussion).

### Experiment Two: Successive Presentation, Variable Edge Polarity

In Experiment Two, we presented two grey rings in rapid succession, rather than simultaneously (Materials and Methods). Subjects first viewed the reference display (one second duration), followed immediately by the matching display (also one second duration). We employed a *similarity rating procedure* in which subjects rated how well the grey shade of the matching ring matched the grey shade of the reference ring (10 = perfect match; 1 = as different as black and white; intermediate values = partial matches of variable quality). We kept the overall contrast the same between the reference and matching displays but varied the relationship between edge polarities. In one condition (Figure 2C), the reference ring was bordered by a black background and a white disk. The subsequent matching ring was bordered by a white background and a black disk. A second condition was composed of the complementary set of polarity relationships (Figure 2D). In two control conditions, the polarity relationships of the reference and matching displays (disks and backgrounds) remained the same over the interval. In each trial, we varied the luminance of the reference ring between the maximum and minimum luminance values associated with the disk and background (see Materials and methods). The luminance of the matching ring remained constant throughout. The aim of the experiment was to determine the luminance value of the reference ring that produces the best color match with the matching ring. Note that subjects did not actually adjust any variables in the experiment, so the terms “reference” and “matching” are used in the heuristic sense in the context of the present experiment.

**Figure 5:**
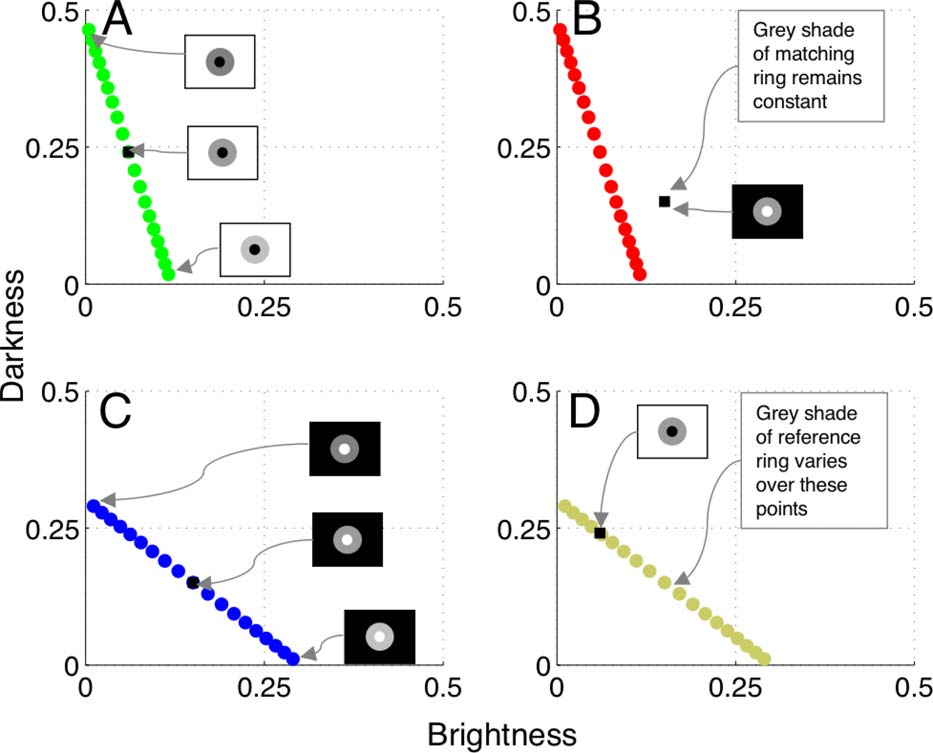
Model Predictions for the Reversed-Polarity Experiment (Experiment Two) The model predicts that subjects will rate ring pairs as identical only when the grey shades associated with reference (colored disks) and matching (black squares) coincide in grey space (A,C). Otherwise, the model predicts that perfect matches will not be possible (B,D). The black square in (D) does not coincide exactly with the position of a yellow disk, although it is very close.

By systematically manipulating edge polarity across reference and matching displays—but keeping the overall contrast the same—we sought to take advantage of the finding in Experiment One that brightness and darkness induction depend on relative edge circumference. All else being equal, the ring-background edge of a given display induced stronger brightness or darkness than the ring-disk edge. This implies that *the achromatic color of the ring changes when edge polarity reverses* across reference and matching displays. Critically, we used the *same ratio of edge circumferences* in Experiments One and Two (Materials and Methods). This allowed us to make a set of theoretical predictions—based on the estimated brightness and darkness weights obtained in Experiment One—to test against the data from Experiment Two. For each of the four conditions of Experiment Two, we inserted the brightness and darkness weights derived from Experiment One into the equations governing the 2-D grey space (Figure 5).

To understand the resultant predictions, consider first a control condition in which the disk and background luminance values are identical for reference and matching displays (Figure 5A). It is clear that the best match (similarity rating = 10) occurs when the reference and matching rings share identical luminance values. In terms of Figure 5A, the black square representing the matching ring coincides exactly with the middle green disk. As the reference- to matching-ring luminance ratio varies in either direction, however, we expect systematic and symmetric deviations from the perfect match. In other words, we expect only partial matches for the remaining green disks in Figure 5A.

A different scenario arises when the polarity relationships between reference and matching rings are reversed. Consider, for example, the black square representing the matching ring in Figure 5B. It is clear that none of the red disks corresponding to the reference rings coincide with the position of the black square. This implies that subjects cannot choose a luminance value of the reference ring to ensure an exact match with the matching ring. The closest possible match, in this case, coincides not with the middle red disk but with one of the disks nearer the bottom of the line of red disks. This observation implies that, in addition to rating the resultant similarity as less than perfect, subjects should choose a luminance value for the reference ring that deviates systematically from the luminance value of the matching ring. The model predicts, in other words, a skewed relationship between similarity ratings and the reference- to matching-ring luminance ratio.

**Figure 6:**
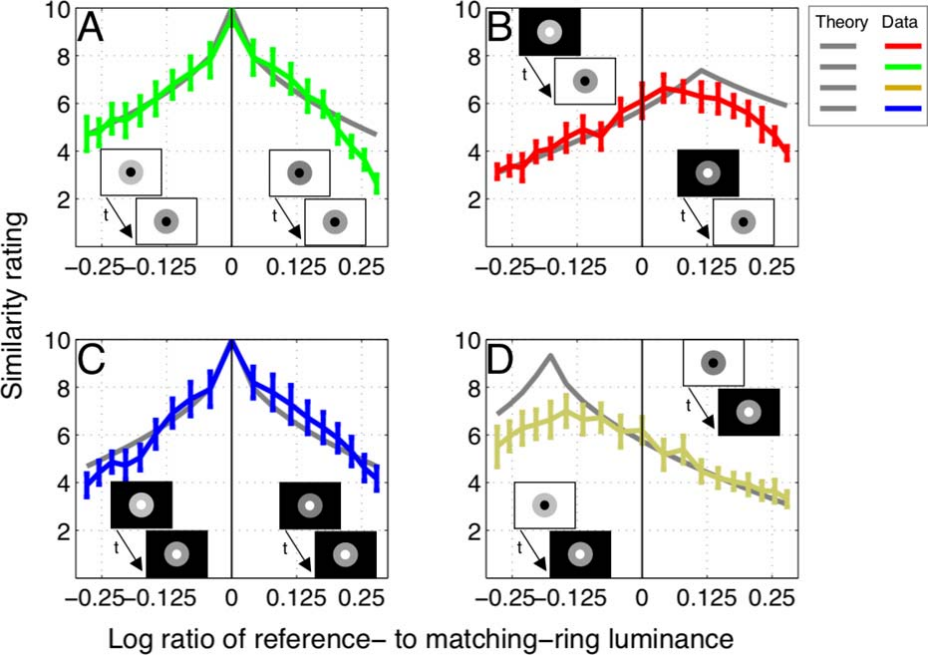
Results of the Reversed-Polarity Experiment (Experiment Two) Average data from six subjects (error bars indicate standard errors of the mean). The model correctly predicts the data curves associated with the two control conditions (A,C). The data curves for the reversed-polarity conditions are consistent with the predicted skewness (B,D), and to a lesser degree with the amount of mismatch.

The mean ratings of six subjects are plotted in Figure 6 along with the model predictions. To a first approximation, the model accurately predicts many aspects of the data. In particular, the model correctly predicts the roughly symmetric rating profiles in the control conditions (Figure 6A and 6C). The model also correctly predicts the skewed nature of the data curves in the reversed-polarity conditions (Figure 6B and 6D). The model does fall down somewhat, however, in predicting the magnitude of the deviation from perfect matches in these conditions. More specifically, the model predicts close to perfect similarity ratings for certain luminance ratios in the condition corresponding to the white reference background and black disk (Figure 6D). The data curve does not support this prediction but rather appears to peak at a level comparable to the peak associated with the curve associated with the black reference background and white disk (see Discussion). In summary, the 2-D model does a reasonable job of quantitatively predicting the results of an experiment differing in several details from the original experiment from which the model was derived.

## Discussion

In Experiment One, we showed that as the contrast difference between reference and matching displays increased, subjects were progressively less able to produce perfect achromatic color matches. Subsequent modeling of these data supported the conclusion that brightness and darkness form perceptual dimensions, with darkness being weighted twice as strongly as brightness. One problem with this interpretation, however, was that decreasing the ring contrast led to a “foggy” appearance resembling transparency [17,18]. Could this fogginess have caused the reported difficulty in making perfect matches? In Experiment Two, we generated stimuli in which overall contrast was high in both reference and match displays. Thus, we reasoned, any difficulty in matching achromatic colors could not be attributable to differences in overall contrast. The similarity ratings of subjects were reasonably consistent with the quantitative predictions of our 2-D model. Perhaps the most important discrepancy was that the model predicted perfect color matches in cases where subjects actually perceived residual color differences. This discrepancy may result from gain control acting on edge signals [4,5,19–21]—which would act to curve nominally straight lines in achromatic color space [6,8]—thereby distorting our estimates of points in grey space. Although we found a small effect of gain control in Experiment One, we chose to omit this factor in our predictions for Experiment Two in order to cap the degrees of freedom of our model. Alternatively, it may be that achromatic color space is composed of even more than two dimensions, with luminance offering a possible candidate for the extra dimension [22,23]. Further experiments are clearly required to resolve this issue.

What might be the functional advantage of the 2-D grey space introduced here? Relative to a 1-D space, the range of achromatic colors available in a 2-D space is enormous. Whereas a 1-D space encodes (n) grey shades (just noticeable differences), a 2-D space contains 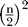 grey shades. Compared to a 1-D space with n = 1,000, for example, the number of discriminable grey shades in a 2-D space equals 250,000. Such a 2-D representation may play a key role in the encoding of achromatic colors in natural environments, where object surfaces form both increments and decrements with respect to variegated backgrounds [24]. The preservation of brightness and darkness information in a 2-D space ensures that the visual system is sensitive to the variance and skewness of luminance pixels bordering an object, rather than just the mean. Such sensitivity may facilitate object detection at low contrasts and play a role in texture discrimination [25–30].

Our framework is consistent with the recent finding that the visual system uses skewness as an image cue to classify bright and dark image regions into “object” and “light” properties [29]. The computation of object and light properties is not, however, required to explain our data. A perceptual space composed of brightness and darkness dimensions explains most of our findings in a parsimonious and quantitative manner. Indeed, our results provide a challenge to anchoring theories of achromatic color perception [10,31] and to the proposal that the dimensions of achromatic color space correspond to object and light properties [6,7]. Several studies have shown that subjects often need to be explicitly instructed to interpret flat, computer-generated displays in terms of object and light properties [21,32,33]. In the present study, subjects were explicitly instructed to match grey shades they saw, rather than putative object or light properties. Importantly, some recent psychophysical findings suggest that judgments of object and light properties may be based on spatial comparisons of surface brightness (and, by extension here, darkness) across different regions of a 3-D scene [34,35]. In our displays, it remains problematic to describe how object and light properties might vary with the difference in contrast magnitude (Experiment One) or polarity (Experiment Two) between reference and matching displays. How, for instance, should one interpret a change in edge polarity between displays? The polarity reversal would not itself seem to carry conflicting information about object and light properties, as both local contrast magnitude and luminance remain the same.

It is well-known that the edges directly bordering a target surface (local edges) and edges distant from the target surface (remote edges) both contribute to achromatic color [3–5,20,21]. The present study helps to clarify the results of our previous study [5] in which we found that subjects had greater difficulty making achromatic color matches with opposite-polarity (relative to same-polarity) combinations of local and remote edges. This puzzle is clarified by noting that opposite-polarity edge combinations simultaneously induce both brightness and darkness into a surface. As in the present study—in which we simultaneously induced brightness and darkness at different local borders—this would lead to a situation in which only partial color matches are possible. Consider, for example, a disk-ring reference configuration in which the local edge induces brightness into the *disk*, whereas the remote edge induces darkness into the disk. In our previous experiment, subjects adjusted a matching disk on a uniform background to appear the same grey shade as the reference disk. This means that the polarity of the disk relative to the background could be either an increment or a decrement. Subjects could therefore match either brightness or darkness, but not both.

It is perhaps illuminating to speculate on the phenomenological aspects of simultaneously perceiving brightness and darkness. Anstis [36] studied the “metallic” or “lustrous” appearance of a surface region composed of both brightness and darkness, generated either through monocular or binocular fusion. The binocular effect is generated by fusion of a dark disk (decrement) presented to one eye with a bright disk (increment) presented to the other eye. The monocular version is obtained by rapidly modulating (in time) the polarity of a disk with respect to a steady background [11]. A third (monocular) version of the effect is generated by embedding a grey disk in a black Ehrenstein pattern on a white background [37]. In all cases, edges of opposite contrast polarity combine to give the overall impression of lustre to the surface. Anstis [36] argued that lustrous surfaces often shimmer, leading him to conclude that lustre is associated with competition between local bright (ON) and dark (OFF) channels [12]. Our results suggest precisely the opposite conclusion. We claim that ON and OFF channels remain separate at the highest levels of processing, giving rise to percepts of simultaneous brightness and darkness, which can be interpreted as appearing lustrous. A weak impression of lustre may be seen in Figure 2A and Figure 2B, where the higher-contrast rings appear “sharper” or more “metallic” than the “softer-appearing” low-contrast rings. This conclusion implies that lustre can be dissociated from shimmer.

How much evidence is there to link the properties of ON and OFF pathways with brightness and darkness perception [11,12]? A recent neurophysiological report reveals that gain control operating in early ON and OFF pathways is sensitive to the variance, but not the skewness and kurtosis, of background pixels [19]. Demonstrations of perceptual sensitivity to skewness [29] thus further highlight the mismatch between the properties of early ON and OFF pathways and achromatic color perception [12,14]. As indicated previously, separate ON and OFF pathways are maintained even at the level of V2 in monkey visual cortex [13]. Researchers have claimed that some neurons in V1 and V2 encode achromatic colors [38–41]. These claims have, however, been disputed [42–44]. To correlate with achromatic color perception in our displays, a visual area would need to combine local edge information into a global surface representation [4,5,21]. The computational nature of this brain area remains to be elucidated.

**Figure 7:**
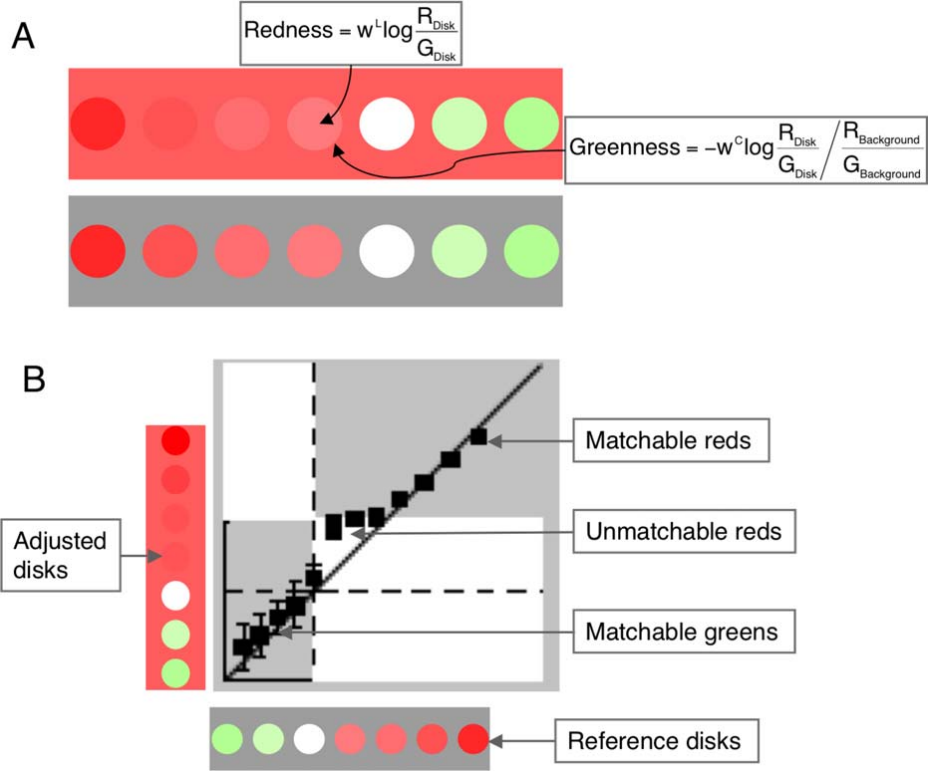
Redness and Greenness as Perceptual Dimensions (A) Stimulus conditions giving rise to mixed color percepts composed of complementary local and edge-induced colors. (B) The *x*- and *y*-axes correspond to the CIE-designated redness and greenness of the reference and matching displays, respectively. The matching (or adjustable) disk is matchable to the reference disk when the reference disk is green (the red matching background adding greenness to the matching disk) or more red than the matching background (the red matching background subtracting redness from the matching disk. No match can be made, however, when the disk is *less red* than the matching background. We claim that this is because the matching background adds greenness to the matching disk, which remains separate from the local redness. As there is no corresponding color induced from the grey reference background into the reference disk, only a partial color match is possible. Data adapted from [18].

As a final twist, we propose that redness and greenness—as well as blueness and yellowness—may form perceptual dimensions [18], rather than canceling [45–47]. Ekroll et al. [18] have shown that subjects can only match certain combinations of local and remotely induced colors (Figure 7). Subjects can, for example, adjust a matching disk on a red background to appear the same hue and saturation as a green reference disk on a grey background. Subjects can also make a suitable match when the *reference disk is a more saturated shade of red than the matching background*. Subjects cannot, however, perform suitable matches when the *reference disk is a shade of red less saturated than the matching background*. Ekroll et al. described similar results for blueness and yellowness perception.

We explain these paradoxical results in terms of the assumption that redness and greenness (blueness and yellowness) form perceptual dimensions. Following Brenner et al. [48], we propose that local color signals (within a surface region) and remote color signals (induced at edges) combine to give rise to the color percept. If the local color signal is red, for example, and the induced color signal is green, then the resultant percept will be composed of both redness and greenness. In the above example, the matching disk *can* be adjusted to match the reference disk *when the reference disk is green*. To understand why, consider a subject attempting to match only the local green color. Matching the local green component alone will not be sufficient because the red matching background also adds greenness to the matching disk. In order to make a perfect match, the subject therefore dials down the physical greenness of the matching disk by an appropriate amount. Similarly, subjects *can* perfectly match the reference disks whose *red shades are more red than the matching background*. Again consider a subject setting a match for the local redness alone. This match will not be perfect because the red matching background subtracts redness from the matching disk. A perfect match thus requires dialing the physical redness of the matching disk up by a certain amount. No match can be made, however, when the reference disk is *less red than the matching background.* Consider a subject matching only the local redness. This requires setting the matching disk as a physical shade of red slightly less saturated than the red of the matching background. The key point here is that the red matching background induces greenness into the match disk. The 2-D model advocated here implies that this induced greenness does not subtract from the local redness. As there is no corresponding greenness induced from the grey reference background into the reference disk, matching only the local redness will *not* give rise to a perfect color match. In such cases, subjects display a wide range of behavioral strategies in a vain attempt to solve an insoluble problem [18].

## Materials and Methods

### Experiment One.

Monitor calibration and experimental methods/procedures were similar to those documented elsewhere [5]. The stimuli consisted of a reference ring of constant luminance (30 cd/m^2^). The starting value of the matching ring varied randomly between 10 and 90 cd/m^2^. The reference disk and background adopted luminance values of either 10 and 90 cd/m^2^ (high contrast) or 25 and 36 cd/m^2^ (low contrast). The matching disk and background adopted luminance values of either 10 and 90, 15 and 60, 20 and 45, or 25 and 36 cd/m^2^. In total, 16 reference-match combinations of these luminance values were used. The diameters of the inner and outer ring edges were respectively 2° and 6°, and the centers of matching and reference disks were 14.4° apart. The gradient in background luminance was restricted to be within 6.2° about the midline of the monitor. Order of stimulus presentation was pseudo-randomized.

### Computational model.

We seek to characterize the residual perceptual difference between reference and matching rings, as follows. Let R, B, D correspond to the luminance values of the ring (R), the bright (B) contiguous surface, and the dark (D) contiguous surface (either disk or background). Following our previous approach [5], we define the respective brightness and darkness signals associated with the ring as 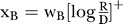 and 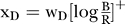 . The parameters w_B_ and w_D_ represent the weights applied to the increment and decrement signals computed in the ON and OFF pathways of early visual processing [11,12]. The operator 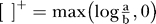 represents half-wave rectification, implying that only non-negative brightness and darkness values are possible. By writing separate equations for matching and reference displays with appropriate scripting (superscripts m and r for match and reference), we define the matching task as the task of setting 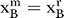 and 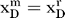 . We solve these equalities separately for logR^m^, giving 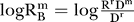 and 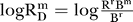 . We then assume that subjects weight the two estimates of logR^m^, giving the generic solution for the log of the matching ring luminance as logR^m^ 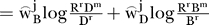 , with the superscript *j* = 1,2 representing the inner and outer edges. We estimated the weights using a nonlinear least-squares optimization routine. Note that we identify the values of the estimated weights with the *theoretical weights* associated with brightness and darkness dimensions, 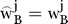 and 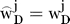 . We adopt this interpretation even though the theoretical weights cancel during calculation of the separate brightness and darkness solutions, log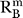 and log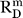 . The argument is that subjects attempt to compromise between perfect darkness and brightness matches by setting log R^m^ between the values log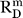 and log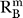 , meaning that we can estimate the *relative brightness and darkness weights*, such that 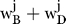 = 1.

We then separately calculated absolute differences between reference and matching rings in the brightness and darkness dimensions of achromatic color space:

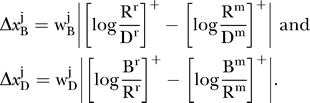



Note that a perfect match is only possible for “identity matches” (B^m^ = B^r^ and D^m^ = D^r^). Match possibility was computed using a modified city-block metric suitable for bounded data, 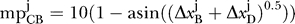 . We also constructed a measure based on the Euclidian metric, 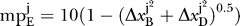 . This measure did not, however, correspond closely with the data, a conclusion consistent with previous results [6]. In practice, we allowed weights to vary linearly with the luminance of the matching background and disk. This factor simulated gain control of edge signals [4,5]. The ensuing model had eight free parameters, two for each of the colored curves in Figure 3. We also tested a model in which the weights remained constant with contrast, giving a total of four free parameters. This model performed only marginally worse. In other words, edge weights did not vary greatly with edge contrast. The results shown in Figure 3 were derived using the more complex model. We assumed throughout the modeling that subjects obeyed one of the constraints, B^m^ > R^m^ > D^m^ or B^m^ < R^m^ < D^m^, depending on the stimulus. This was true for all but the leftmost data point in Figure 4C. All computations were performed in Matlab (version 7.0.4, The MathWorks) using standard and customized functions.

### Experiment Two.

The design of Experiment Two was the same as that of Experiment One, except for the changes below, which were tailored to counter specific criticisms of the reviewers. The changes were as follows: (1) the reference and matching rings were presented in rapid sequence, rather than simultaneously, in order to minimize the role of eye movements and adaptation (simultaneous presentation would also have required a very steep, therefore noticeable, luminance gradient across the screen); (2) the subjects' task was to *judge the similarity in grey shades* of the successive rings, thereby minimizing the use of elaborate strategies during a matching task. Even though there was no matching task, we adopt the nomenclature of calling the first display the reference and the second the matching display; (3) the polarity of the reference and matching disks and backgrounds either stayed the same or flipped in polarity, whereas the total contrast, log 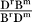 , remained constant throughout; (4) in each trial, the luminance of the reference ring had one of 19 luminance values, defined as constant steps in log space between the minimum and maximum values of the disk and background (15 and 60 cd/m^2^). The value of the matching ring always remained constant at 30 cd/m^2^. The experiment had four conditions, with trials being randomized across conditions. In the two control conditions, the polarity of the disks and backgrounds stayed the same (D^r^ = D^m^ = 60, B^r^ = B^m^ = 15, D^r^ = D^m^ = 15, B^r^ = B^m^ = 60). In the other two conditions, the polarity of the disks and backgrounds flipped (D^r^ = B^m^ = 60, B^r^ = D^m^ = 15, B^r^ = D^m^ = 15, D^r^ = B^m^ = 60). Subjects performed one entire run of the experiment in order to generate an internal scale for the similarity ratings. The ratio of diameters of the inner and outer ring edges was the same as in Experiment One (1:3). The rings were, however, much larger than in Experiment One, with the ring and disk subtending approximately 3.5° and 10.5°, respectively.

### Proof: 1-D space implies perfect achromatic color matching.

Letting x_B_ = w_B_ 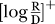 and x_D_ = w_D_ 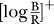 again, assume that the early brightness and darkness signals subtract at a cortical processing stage [13], then *net* brightness and darkness signals are computed as Ψ_B_ = [X_B_ − X_D_]^+^ and Ψ_D_ = [X_D_ − X_B_]^+^. Since Ψ_B_ and Ψ_D_ cannot be simultaneously positive, all possible grey shades can only contain either brightness or darkness, but not both at the same time. Thus, all grey shades can be specified by a single number, as seen most easily by removing the half-wave rectification constraint on Ψ_B_ and Ψ_D_, implying that Ψ_B_ = − Ψ_D_ and −Ψ_B_ = Ψ_D_. Thus the corollary of the subtraction assumption is that all possible grey shades are contained within a single dimension. To see how a 1-D space implies perfect matching, let Ψ_r_ and Ψ_m_ equal either the net brightness or darkness associated with the reference and matching rings, respectively. Letting Ψ_m_ = Ψ_r_, we derive logR^m^ = 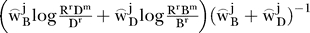 . The existence of this solution shows that the luminance of the matching ring can always be set to achieve a perfect match. We have confirmed this result using a 1-D model of the data presented here.

### Calculation of darkness–brightness weight ratio.

Let the *true* darkness–brightness weight ratio be represented by 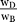 . We know the two circumference-biased estimates, 
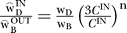
 and 
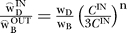
 . Thus, 
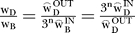
 . We solve, n = log 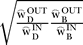 /log(3) ≈ log 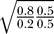 /log(3) ≈ 0.63, giving 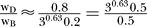 ≈2.

## References

[pcbi-0030179-b001] Wallach H (1948). Brightness constancy and the nature of achromatic colors. J Exp Psych.

[pcbi-0030179-b002] Grossberg S, Todorovic D (1988). Neural dynamics of 1-D and 2-D brightness perception: A unified model of classical and recent phenomena. Percept Psychophys.

[pcbi-0030179-b003] Arrington KF (1996). Directional filling-in. Neural Comput.

[pcbi-0030179-b004] Rudd ME, Zemach IK (2004). Quantitative properties of achromatic color induction: An edge integration analysis. Vision Res.

[pcbi-0030179-b005] Vladusich T, Lucassen MP, Cornelissen FW (2006). Edge integration and the perception of brightness and darkness. J Vis.

[pcbi-0030179-b006] Logvinenko AD, Maloney LT (2006). The proximity structure of achromatic surface colors and the impossibility of asymmetric lightness matching. Percept Psychophys.

[pcbi-0030179-b007] Heggelund P (1992). A bidimensional theory of achromatic color vision. Vision Res.

[pcbi-0030179-b008] Izmailov CA, Sokolov EN (1991). Spherical model of color and brightness discrimination. Psychol Sci.

[pcbi-0030179-b009] Foster DH (2003). Does colour constancy exist?. Trends Cogn Sci.

[pcbi-0030179-b010] Gilchrist A, Kossyfidis C, Bonato F, Agostini T, Cataliotti J (1999). An anchoring theory of lightness perception. Psychol Rev.

[pcbi-0030179-b011] Magnussen S, Glad A (1975). Brightness and darkness enhancement during flicker: Perceptual correlates of neuronal B- and D-systems in human vision. Exp Brain Res.

[pcbi-0030179-b012] Schiller PH (1992). The ON and OFF channels of the visual system. Trends Neurosci.

[pcbi-0030179-b013] Wang Y, Xiao Y, Felleman DJ (2007). V2 thin stripes contain spatially organized representations of achromatic luminance change. Cereb Cortex.

[pcbi-0030179-b014] Rossi AF, Paradiso MA (1999). Neural correlates of perceived brightness in the retina, lateral geniculate nucleus, and striate cortex. J Neurosci.

[pcbi-0030179-b015] Hamada J (1985). Asymmetric lightness cancellation in Craik-O'Brien patterns of negative and positive contrast. Biol Cybern.

[pcbi-0030179-b016] De Weert CM, Spillmann L (1995). Assimilation: Asymmetry between brightness and darkness?. Vision Res.

[pcbi-0030179-b017] Anderson BL, Winawer J (2005). Image segmentation and lightness perception. Nature.

[pcbi-0030179-b018] Ekroll V, Faul F, Niederee R (2004). The peculiar nature of simultaneous colour contrast in uniform surrounds. Vision Res.

[pcbi-0030179-b019] Bonin V, Mante V, Carandini M (2006). The statistical computation underlying contrast gain control. J Neurosci.

[pcbi-0030179-b020] Rudd ME, Arrington KF (2001). Darkness filling-in: A neural model of darkness induction. Vision Res.

[pcbi-0030179-b021] Rudd ME, Zemach IK (2005). The highest luminance anchoring rule in achromatic color perception: Some counterexamples and an alternative theory. J Vis.

[pcbi-0030179-b022] Shapiro AG, D'Antona AD, Charles JP, Belano LA, Smith JB (2004). Induced contrast asynchronies. J Vis.

[pcbi-0030179-b023] Shapiro AG, Charles JP, Shear-Heyman M (2005). Visual illusions based on single-field contrast asynchronies. J Vis.

[pcbi-0030179-b024] Frazor RA, Geisler WS (2006). Local luminance and contrast in natural images. Vision Res.

[pcbi-0030179-b025] Arend LE, Spehar B (2004). Mechanisms of contrast induction in heterogeneous displays. Vision Res.

[pcbi-0030179-b026] Brown RO, MacLeod DI (1997). Color appearance depends on the variance of surround colors. Curr Biol.

[pcbi-0030179-b027] Chubb C, Nam J (2000). Variance of high contrast textures is sensed using negative half-wave rectification. Vision Res.

[pcbi-0030179-b028] De Bonet JS, Zaidi Q (1997). Comparison between spatial interactions in perceived contrast and perceived brightness. Vision Res.

[pcbi-0030179-b029] Motoyoshi I, Nishida S, Sharan L, Adelson EH (2007). Image statistics and the perception of surface qualities. Nature.

[pcbi-0030179-b030] Spehar B, Debonet JS, Zaidi Q (1996). Brightness induction from uniform and complex surrounds: A general model. Vision Res.

[pcbi-0030179-b031] Bressan P (2006). The place of white in a world of grays: A double-anchoring theory of lightness perception. Psychol Rev.

[pcbi-0030179-b032] Arend LE, Spehar B (1993). Lightness, brightness, and brightness contrast: 1. Illuminance variation. Percept Psychophys.

[pcbi-0030179-b033] Arend LE, Spehar B (1993). Lightness, brightness, and brightness contrast: 2. Reflectance variation. Percept Psychophys.

[pcbi-0030179-b034] Robilotto R, Zaidi Q (2004). Limits of lightness identification for real objects under natural viewing conditions. J Vis.

[pcbi-0030179-b035] Robilotto R, Zaidi Q (2006). Lightness identification of patterned three-dimensional, real objects. J Vis.

[pcbi-0030179-b036] Pinna B, Spillmann L, Ehrenstein WH (2002). Scintillating lustre and brightness induced by radial lines. Perception.

[pcbi-0030179-b037] Anstis SM (2000). Monocular lustre from flicker. Vision Res.

[pcbi-0030179-b038] Kinoshita M, Komatsu H (2001). Neural representation of the luminance and brightness of a uniform surface in the macaque primary visual cortex. J Neurophysiol.

[pcbi-0030179-b039] Roe AW, Lu HD, Hung CP (2005). Cortical processing of a brightness illusion. Proc Natl Acad Sci U S A.

[pcbi-0030179-b040] Rossi AF, Rittenhouse CD, Paradiso MA (1996). The representation of brightness in primary visual cortex. Science.

[pcbi-0030179-b041] Boyaci H, Fang F, Murray SO, Kersten D (2007). Responses to lightness variations in early human visual cortex. Curr Biol.

[pcbi-0030179-b042] Cornelissen FW, Vladusich T (2006). What gets filled-in during filling-in?. Nat Rev Neurosci.

[pcbi-0030179-b043] Cornelissen FW, Wade AR, Vladusich T, Dougherty RF, Wandell BA (2006). No functional magnetic resonance imaging evidence for brightness and color filling-in in early human visual cortex. J Neurosci.

[pcbi-0030179-b044] Vladusich T, Lucassen MP, Cornelissen FW (2006). Do cortical neurons process luminance or contrast to encode surface properties?. J Neurophysiol.

[pcbi-0030179-b045] Hsieh PJ, Tse PU (2006). Illusory color mixing upon perceptual fading and filling-in does not result in “forbidden colors.”. Vision Res.

[pcbi-0030179-b046] Hurvich LM, Jameson D (1974). Opponent processes as a model of neural organization. Am Psychol.

[pcbi-0030179-b047] Miyahara E, Smith VC, Pokorny J (2001). The consequences of opponent rectification: The effect of surround size and luminance on color appearance. Vision Res.

[pcbi-0030179-b048] Brenner E, Granzier JJ, Smeets JB (2007). Combining local and global contributions to perceived colour: An analysis of the variability in symmetric and asymmetric colour matching. Vision Res.

